# Pediatric ABCC6 deficiency: a genotypic and phenotypic analysis

**DOI:** 10.1186/s13023-025-04102-7

**Published:** 2025-11-19

**Authors:** Marta Bertamino, David J. Goldberg, M. Zulf Mughal, Lisa Pabst, Yaping Joyce Liao, Lisa R. Sun, Jane Beckwell, Amina Kozaric, Ruth du Moulin, Katie Swanner, Carlos R. Ferreira, Shira G. Ziegler

**Affiliations:** 1 Physical Medicine and Rehabilitation Unit, IRCCS Instituto Giannina Gaslini, Genoa, Italy; 2 Division of Cardiology, Department of Pediatrics, Children’s Hospital of Philadelphia, Philadelphia, PA USA; 3 Department of Pediatric Endocrinology, Al Jalila Children’s Specialty Hospital, Dubai, UAE; 4 Division of Neurology, Department of Pediatrics, University of Utah, Salt Lake City, UT USA; 5 Department of Ophthalmology, Stanford University School of Medicine, Stanford, CA USA; 6 Department of Neurology, Stanford University School of Medicine, Stanford, CA USA; 7 Division of Pediatric Neurology, Division of Stroke, Department of Neurology, Johns Hopkins University School of Medicine, Baltimore, MD USA; 8 Genomenon, Inc, Ann Arbor, MI USA; 9 Inozyme Pharma, Boston, MA USA; 10 Eunice Kennedy Shriver National Institute of Child Health and Human Development, National Institutes of Health, Bethesda, MD USA; 11 Department of Genetic Medicine, Johns Hopkins University School of Medicine, Baltimore, MD USA

**Keywords:** ABCC6 deficiency, GACI2, PXE, Clinical phenotype, Genotype

## Abstract

**Background:**

ABCC6 deficiency is caused by variants in the *ABCC6* gene, leading to dysfunction of the ABCC6 protein. This can result in the development of the infantile phenotype, generalized arterial calcification of infancy type 2 (GACI2), or the adolescent-adult phenotype, pseudoxanthoma elasticum (PXE). To date, the impact of ABCC6 deficiency in a pediatric population has not been comprehensively studied. This analysis aimed to collectively characterize the genotypic and phenotypic presentation of ABCC6 deficiency in the pediatric population.

**Results:**

A literature review and analysis identified 95 individuals with *ABCC6* variant(s) and documented clinical manifestations occurring from ages 0 to < 18 years. Of the 133 *ABCC6* variants found, 57.1% were pathogenic, 26.3% were likely pathogenic, and 10.5% were of uncertain significance. A high prevalence of ectopic calcification with cardiovascular, dermatologic, neurologic, and ocular complications was observed across this pediatric ABCC6 deficiency cohort. While 56% were diagnosed with GACI and 44% with PXE, many individuals exhibited overlapping features of both conditions. There was a relatively high frequency of clinical manifestations through 6 years of age with lower frequency from ages 7 to 18 years. There was significant phenotypic variability observed across patients harboring the same *ABCC6* variant(s).

**Conclusions:**

These findings demonstrate the wide spectrum and early emergence of cardiovascular, neurologic, and ocular complications in pediatric patients with ABCC6 deficiency. Given the variability of clinical presentations and absence of systematic phenotype characterization, pediatric ABCC6 deficiency is likely underdiagnosed. Establishing guidelines for assessment, genetic diagnosis, monitoring, and prognostic counseling would assist in the timely diagnosis and multidisciplinary management of pediatric patients with ABCC6 deficiency.

**Supplementary Information:**

The online version contains supplementary material available at 10.1186/s13023-025-04102-7.

## Background

Adenosine triphosphate (ATP) binding cassette subfamily C member 6 (ABCC6) is a transmembrane protein predominantly expressed in the basolateral plasma membrane of hepatocytes and, to a lesser extent, in the proximal tubules of the kidney [1–6]. There, ABCC6 is involved in extracellular ATP efflux and metabolism, though its exact function remains unknown. Extracellular ATP serves as a substrate for production of inorganic pyrophosphate (PP_i_) and adenosine monophosphate (AMP) by ectonucleotide pyrophosphatase/phosphodiesterase 1 (ENPP1) [2]. AMP is further metabolized by CD73 into adenosine and inorganic phosphate (P_i_) [5, 7]. This process plays a critical role in the homeostasis of calcification by regulating extracellular levels of PP_i_, which acts as a potent inhibitor of pathological mineralization [2]. Dysfunction of the ABCC6 protein results in a reduction in extracellular ATP, creating an imbalance in PP_i_ levels that leads to ectopic calcification, while reduced AMP and adenosine contribute to arterial intimal proliferation and narrowing of the arterial lumen [1, 2, 5, 7].

Over 300 *ABCC6* variants have been identified, but only 35% are currently recognized as contributors to disease [8]. To date, *ABCC6* variants have been associated with two disease phenotypes: generalized arterial calcification of infancy type 2 (GACI2; OMIM #614473) and pseudoxanthoma elasticum (PXE; OMIM #264800) [3, 4]. The prevalence of ABCC6 deficiency, based on variants occurring in PXE, is estimated to be 1:25,000 to 1:50,000 people [5, 9].

GACI typically presents prenatally or in early infancy; it is characterized by pathological calcifications in medium-to-large–sized arteries and intimal proliferation leading to vascular occlusion and related sequelae of ischemia, including myocardial infarction, heart failure, and stroke [4, 10]. The majority of cases are caused by pathogenic variants in *ENPP1* (GACI1), and approximately 9% of cases are attributed to variants in *ABCC6* (termed GACI2) [11]. Mortality rates for GACI2 have previously been estimated at 10.5% of affected infants; although this is lower than the mortality rate for GACI1 (40.5% mortality), it remains life-threatening [12]. In those who survive beyond infancy, the prognosis has not been well characterized, but case reports show that individuals may continue to experience cardiovascular and/or neurologic complications [13–19].

PXE is considered the primary phenotype of ABCC6 deficiency. Compared with GACI2, PXE has a later onset in adolescence to adulthood and is characterized by ectopic mineralization of elastic fibers, primarily in the skin, Bruch’s membrane of the eyes, and the cardiovascular system [3–5, 20–22]. A definitive diagnosis of PXE can be made if two or more major criteria from different categories of the established PXE diagnostic criteria are met. These categories include skin or eye manifestations, or a genetic test confirming biallelic pathogenic variants in *ABCC6* [22, 23].

GACI2 and PXE share significant phenotypic overlap. Both conditions commonly involve vascular mineralization, and while infants often present with severe heart failure or hypertension, adults with PXE have increased risk of peripheral arterial disease and ischemic stroke [21, 23–26]. In a recent analysis of 19 GACI2 patients, 21% had a history of stroke [12]. Notably, some carriers of a single *ABCC6* variant may also have a clinically relevant phenotype, including retinal alterations, aberrant lipid metabolism, or peripheral arterial disease [27–29]. Additionally, several heterozygous *ABCC6* variants are an established risk factor for ischemic stroke [30] and are included on pediatric stroke gene panels [31]. It is unknown if these clinical manifestations are related to true haploinsufficiency or if a second *ABCC6* variant is present in an unsequenced deep intronic region of the second allele [32, 33].

The impact of ABCC6 deficiency between infancy and adulthood is poorly understood. To date, the clinical manifestations associated with *ABCC6* variants in the pediatric population come mostly from case reports and have not been comprehensively analyzed. Therefore, the aim of this analysis was to elucidate the genotypic and phenotypic spectrum of ABCC6 deficiency in the pediatric population.

## Methods

A descriptive analysis of individuals with ABCC6 deficiency and clinical complication(s) reported between ages 0 to < 18 years was conducted. ABCC6 deficiency was defined as one or more ABCC6 variants. Data were integrated from a comprehensive, retrospective literature review along with results from two natural history studies: “Study of People with GACI or ARHR2” (identifier: NCT03478839), performed at the US National Institutes of Health (NIH), and “Natural History of GACI With or Without ARHR2 or PXE” (identifier: NCT03758534), performed at Münster University Children’s Hospital in Germany [12]. The literature review was performed using the data content in Mastermind (Genomenon), a genomic intelligence platform for curated variant data and literature evidence [34], and considered all publications indexed from PubMed as of October 10, 2024. The inclusion criteria for this review were patients with one or more reported variants in *ABCC6*, and any reported clinical phenotype before age 18. In addition, three cases reporting pediatric ABCC6 deficiency without *ABCC6* variants explicitly noted were identified and included in this analysis.

**Fig. 1:**
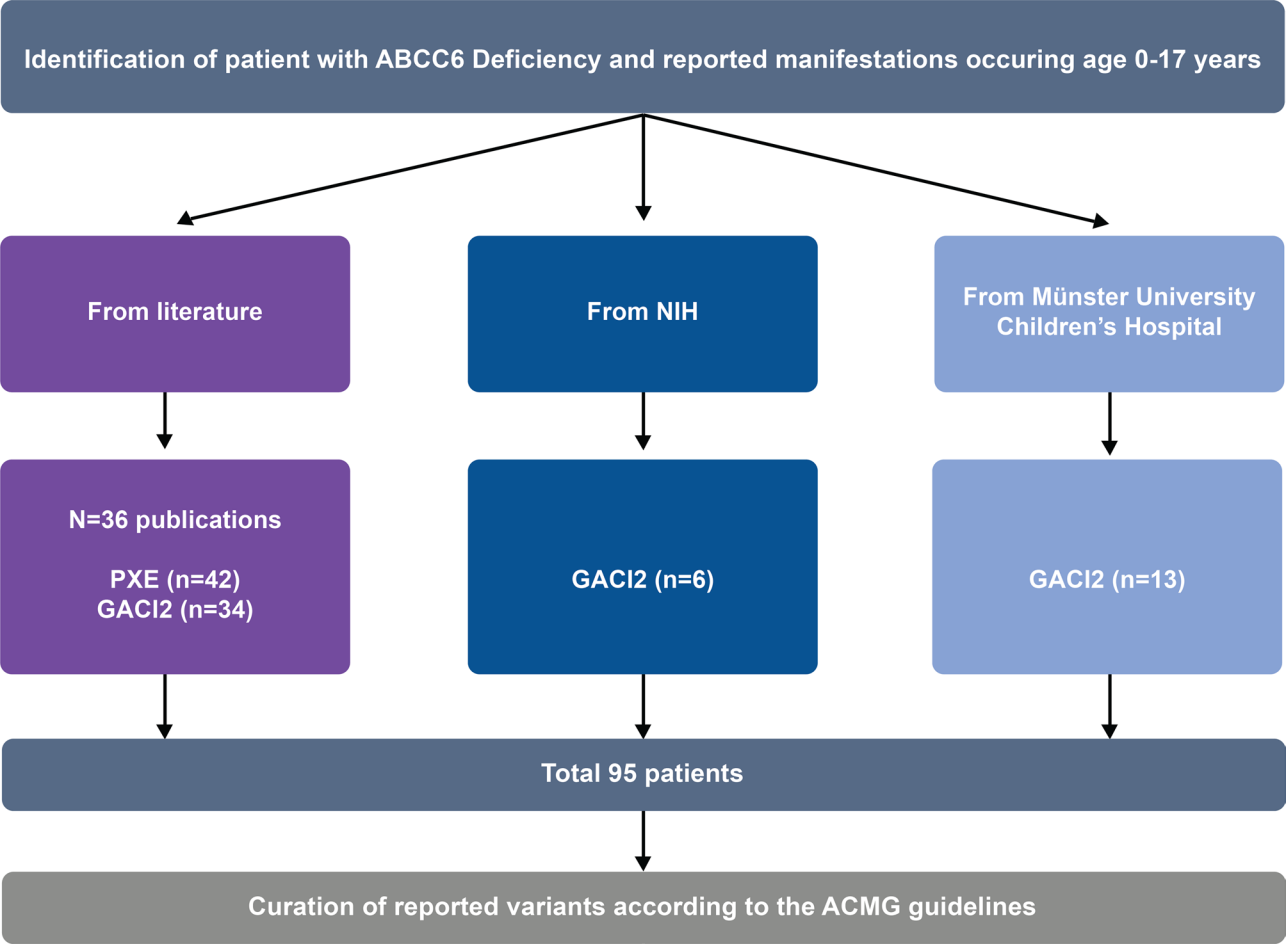
Methodology flow chart

A total of 995 articles citing the *ABCC6* gene symbol and/or associated disease were reviewed. In total, 95 individuals met the inclusion criteria (76 from published literature and 19 from natural history studies) and were included in the analysis set (Fig. 1, Additional file 1, Table S1). Data from each source were reviewed for consistent nomenclature, and duplicate entries were removed; variants were annotated based on clinical and biochemical phenotypes. Variant interpretation was based on the American College of Medical Genetics and Genomics and Association for Molecular Pathology (ACMG/AMP) guidelines [35].

## Results

### Patient demographics and diagnoses

Of the identified 95 individuals with confirmed *ABCC6* variant(s) and clinical manifestations reported between infancy and adolescence, 53 (55.8%) had a reported diagnosis or phenotype consistent with GACI2, and 42 (44.2%) had PXE. Biallelic *ABCC6* variants were identified in 61.0% of the analysis population, while 27.4% had a single identified *ABCC6* variant (Table 1). Across the overall population with available data, the median age of onset, defined as the age at which first symptom/manifestation occurred, was 0.38 years (~ 4.6 months) (range 0 to < 18 years), and the median age at diagnosis was 2.75 years (range 0–50 years). Eleven patients (11.6%) had died at a median age of 0.15 years (~ 1.8 months) (range 0–10 years)—all of whom had been diagnosed with GACI2; survival outcome was not reported for 11 patients.

**Table 1:** Patient demographics

	Total Patients (*n* = 95)	GACI (*n* = 53)	PXE (*n* = 42)
**Patient age, median (range), years**			
At onset^a^	0.38 (0 to < 18)	0 (0-5.25)	7.5 (0–17)
Prenatal, n (%)	6 (6.3)	6 (11.3)	0 (0)
0–2, n (%)	36 (37.9)	34 (56.6)	6 (14.3)
3–4, n (%)	6 (6.3)	3 (5.7)	3 (7.1)
5–7, n (%)	4 (4.2)	1 (1.9)	3 (7.1)
8–10, n (%)	6 (6.3)	0 (0)	6 (14.3)
11–17, n (%)	6 (6.3)	0 (0)	6 (14.3)
Unknown, n (%)	33 (34.7)	15 (28.3)	18 (42.9)
At diagnosis^b^	2.75 (0–50)	0.04 (0-3.5)	11 (2–50)
At death^c^	0.15 (0–10)	0.15 (0–10)	N/A^d^
Alive, n (%)	84 (88.4)	42 (79.2)	42 (100)
Dead, n (%)	11 (11.6)	11 (20.8)	0 (0)
Unknown, n (%)	18 (18.9)	0 (0)	18 (42.9)
**Biallelic ABCC6 variants, n (%)**	58 (61.0)	28 (52.8)	30 (71.4)
Homozygous (all), n (%)	25 (26.3)	6 (11.3)	19 (45.2)
Homozygous P/LP, n (%)	21 (22.1)	6 (11.3)	15 (35.7)
Compound heterozygous (all), n (%)	33 (34.7)	22 (41.5)	11 (26.2)
Compound heterozygous, both P/LP, n (%)	23 (24.2)	16 (30.2)	7 (16.7)
**Single allele (all), n (%)**	26 (27.4)	17 (32.1)	9 (21.4)
Single allele P/LP, n (%)	16 (21.0)	10 (18.9)	6 (12.3)
**Unknown/ no variants, n (%)**	11 (11.6)	8 (15.1)	3 (7.1)

^a^ age of onset data was available for 62% of individuals

^b^ age at diagnosis data was available for 55% of individuals

^c^ age of death data was available for 11 (11.6%) of individuals

^d^ No data. All individuals had an unknown entry

Individuals diagnosed with GACI2 (*n* = 53) predominantly presented with signs/symptoms of disease in infancy (median age of 0 years), with median age at diagnosis of 0.04 years (~ 0.5 months; Table 1). For individuals diagnosed with PXE (*n* = 42), the median age at onset was 7.5 years, with broad distribution from infancy through adolescence, and the median age at diagnosis was 11 years.

### Phenotypes

#### Prevalence of clinical manifestations

Soft tissue calcification was reported in 55.8% of the overall population, with 47.7% and 27.4% of individuals with arterial and organ calcification (excluding skin calcification), respectively (Table 2). Cardiovascular complications were reported in 51.6% of individuals, including systemic hypertension (28.4%), left ventricular hypertrophy (22.1%), signs of heart failure (22.1%), and respiratory distress (17.9%). Skin manifestations, reported as skin lesions (14.7%), papules (8.4%), and plaques (4.2%), were observed in 38.9% of individuals, typically affecting the flexural regions such as the neck, axilla, and knee. Ocular manifestations were reported in 23.1% of individuals and included vision loss (6.3%), visual impairment (5.3%), angioid streaks (5.3%), optic nerve atrophy (3.1%), and retinopathy (3.1%). Neurologic manifestations were reported in 23.1% of individuals, recorded as neurodevelopmental delay (12.6%), stroke (9.4%), seizure or epilepsy (9.4%), transient ischemic attack (TIA) (5.3%), and encephalopathy (2.1%). Five patients (5.2%) had renal dysfunction, including 3 with renal failure. Surprisingly, eight individuals, all diagnosed with GACI2, had a history of metaphyseal changes (*n* = 6) and/or hypophosphatemic rickets (*n* = 3) (Table 2), although serum phosphate levels were not reported. Among these eight patients, seven had a history of bisphosphonate use, and one also carried a variant in *ENPP1*. Only one patient had rachitic changes in the absence of prior bisphosphonate use; this patient had a single heterozygous change in *ABCC6* (c.1769 C > T), a variant with conflicting classification of pathogenicity per ClinVar.

**Table 2:** Prevalence of clinical phenotypes and selected medical events

Phenotype and Clinical Manifestations	Total Individuals *N* = 95	
	*N*	%
**Calcification** ^a^	**53**	**55.8**
Arterial	45	47.7
Organ	26	27.4
Periarticular	6	6.3
**Cardiovascular phenotype**	**49**	**51.6**
Hypertension	27	28.4
Left ventricular hypertrophy	21	22.1
Heart failure	21	22.1
Respiratory distress	17	17.9
Cardiomegaly	13	13.7
Heart valve defect	11	11.5
Cardiomyopathy	8	8.4
Cardiac arrhythmia	6	6.3
Pericardial effusion	6	6.3
Myocardial infarction/cardiac arrest	5	5.3
Heart murmur	4	4.2
**Skin phenotype**	**37**	**38.9**
Skin lesions	14	14.7
Papules	8	8.4
Plaques	4	4.2
**Ocular phenotype**	**22**	**23.1**
Vision loss	6	6.3
Visual impairment	5	5.3
Angioid streaks	5	5.3
Optic nerve atrophy	3	3.1
Retinopathy	3	3.1
**Neurologic phenotype**	**22**	**23.1**
Neurodevelopmental delay	12	12.6
Stroke	9	9.4
Seizure/epilepsy	9	9.4
Transient ischemic attack (TIA)	5	5.3
Encephalopathy / encephalomalacia	2	2.1
**Arterial stenosis**	**21**	**22.1**
**Other**	**14**	**14.7**
Metaphyseal changes / rickets	8	8.4
Renal dysfunction / failure	5	5.2
**Hospitalization (all cause)**	**24**	**25.2**
**Mortality**	**11**	**11.6**

^a^ Excludes skin calcification: Other clinical manifestations in each phenotype category:​

• Cardiovascular: patent ductus arteriosus, cardiac ischemia, decreased cardiac contractility, mid-aortic syndrome, patent ductus arteriosus, patent foramen ovale, right ventricular hypertrophy, atrial septal aneurism, long QT syndrome, coartication of aorta, weakened pulse, tachycardia, bradycardia, chest pain, cardiac hypokinesis, endocardial fibroelastosis, dilated aorta/artery

• Ocular: peau d’orange, choroidal neovascularization, retinal hemorrhage, strabismus, papilledema, nyctalopia

• Neurologic: hydrocephalus, hypotonia, pseudotumor cerebri, ventriculomegaly, spastic tetraparesis, headache, dizziness, migraine

#### Clinical manifestations by age

**Fig. 2:**
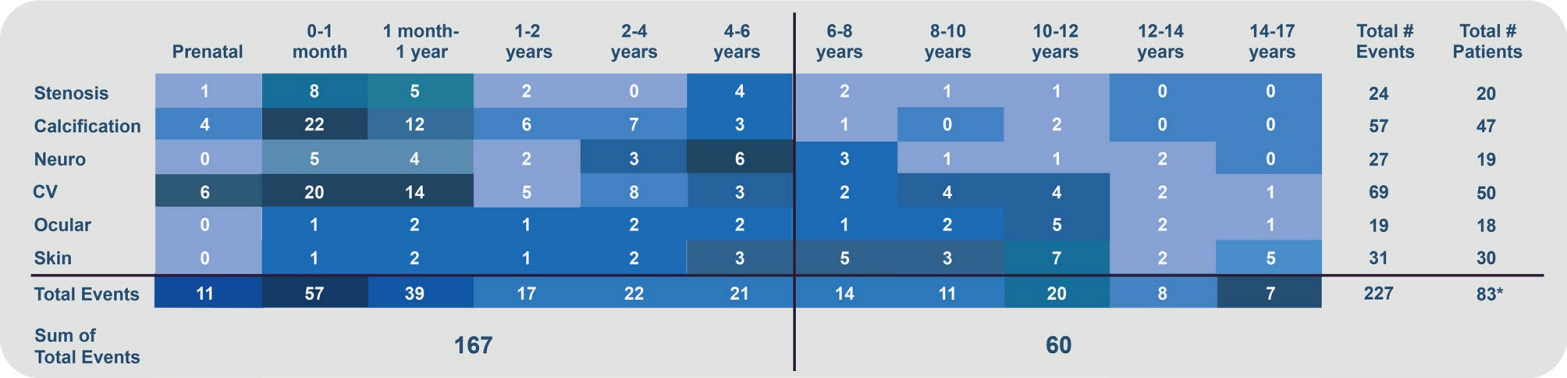
Clinical manifestations by patient age at which they were reported to be present, with sum total of events occurring age ≤ 6 and > 6 years. *12 patients were excluded from analysis becuase exact age at manifestation(s) was not reported

In addition to the median age of disease onset (Table 1), complications based on the age at which they were reported were characterized, providing a comprehensive view of organ system involvement from the prenatal period through adolescence (Fig. 2). When multiple complications within the same organ system were reported simultaneously (e.g., hypertension and heart failure), only one “cardiovascular event” was accounted for at that time point. A total of 227 unique clinical events were identified among 83 individuals (Fig. 2), where 12 patients without specific event ages were excluded. The bulk of events (*n* = 96; 42%) occurred between birth and one year of age, with complications continuing at a relatively high frequency through six years (74% of events) and with lower frequency from age 7 to 17 years (26% of events). Arterial and organ calcifications were recorded prenatally in four individuals, with most calcification observed between birth and one year (60%), and with reduced frequency beyond age 4. Similarly, cardiovascular manifestations were most common in infancy but persisted in older children through age 17. Neurologic complications were reported consistently from infancy to age 8, and to a lesser extent through age 14. In contrast, ocular and skin manifestations were uncommon in infancy, with the majority of events occurring in children over two years, and (skin manifestations especially) becoming more apparent with older age.

#### Individual phenotypes

**Fig. 3:**
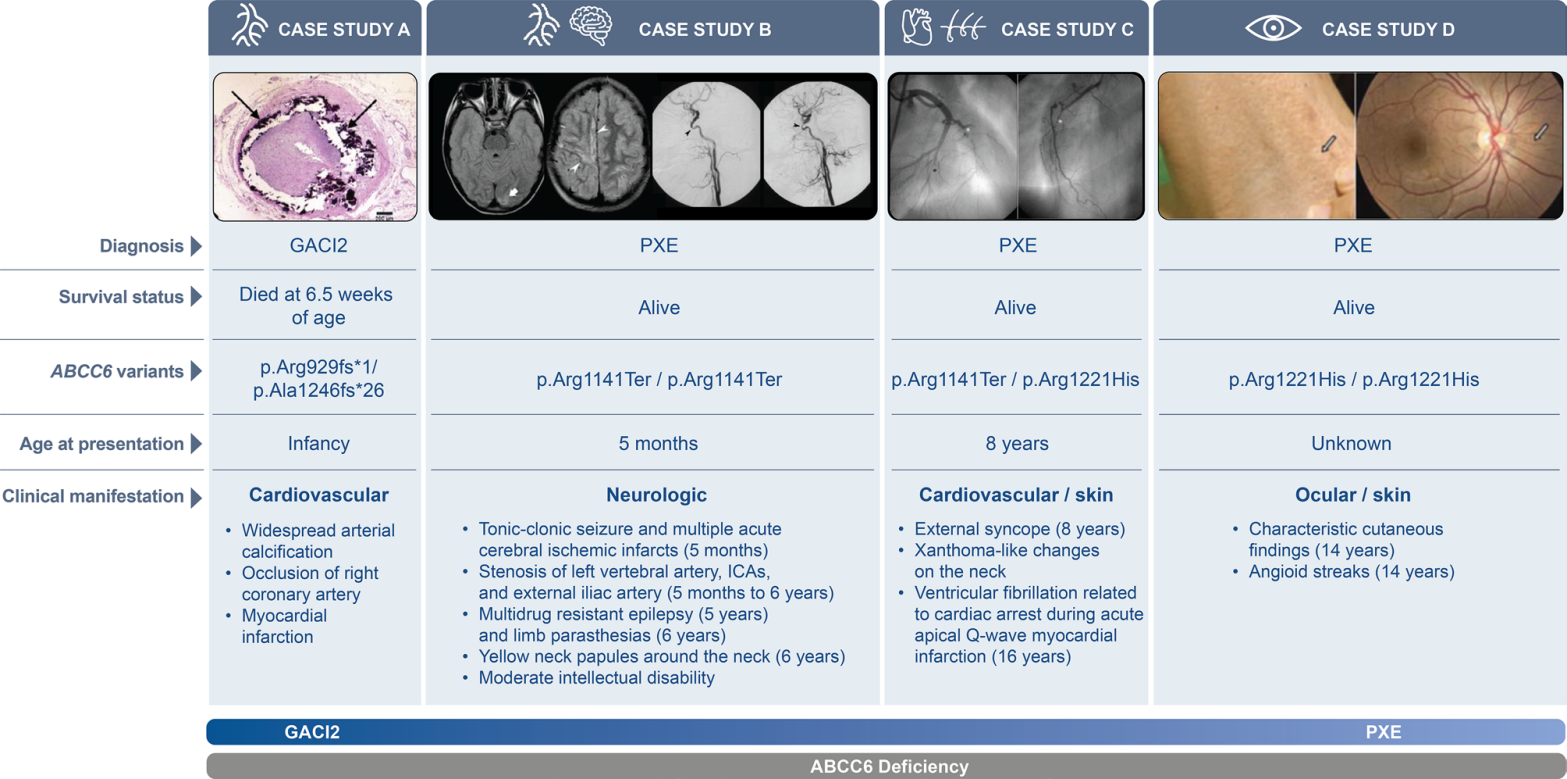
Four published cases of patients with ABCC6 deficiency (defined as one or more ABCC6 variants) and their clinical complications. Case study **A**) Histological detail showing cross section of a coronary artery with disruption of the calcified internal elastic lamina (arrows) and large intima proliferation (hematoxylin and eosin staining, 32.5). Used with permission of Elsevier Science and Technology Journals, from Generalized Arterial Calcification of Infancy and Pseudoxanthoma Elasticum Can Be Caused by Mutations in Either ENPP1 or ABCC6 by Nitschke et al., 90, 2012; permission conveyed through Copyright Clearance Center, Inc [13]. Case study **B**) Neuroradiological detail illustrating first two images: Axial FLAIR images show multiple chronic corticosubcortical infarcts in the left occipital (arrowhead) and right fronto-mesial and precentral regions (thick arrows). Note prominent leptomeningeal collaterals resulting in increased signal intensity within the subarachnoid spaces in the left fronto-parietal regions. Third and fourth images: Lateral digital subtraction angiography (DSA) views of the right internal carotid artery (ICA; third image) and left ICA (fourth image) reveal bilateral narrowing of the petrous segments (arrowheads). Used with permission of Elsevier Science and Technology Journals, from ABCC6 Mutations and Early Onset Stroke: Two Cases of a Typical Pseudoxanthoma Elasticum, by Bertamino et al., 22, 2018; permission conveyed through Copyright Clearance Center, Inc [14]. Case study **C**) An occlusion in the middle segment of the left circumflex coronary artery (black asterisk) and a 99% stenosis in the middle segment of the left anterior descending coronary artery (white asterisk). Reprinted from International Journal of Cardiology, 116, Acute Myocardial Infarction and a New ABCC6 Mutation in a 16-year-old Boy with Pseudoxanthoma Elasticum, Kiec-Wilk et al. 2007, with permission from Elsevier [38]. Case study **D**) Clinical detail depicting cutaneous lesions on side of the neck characteristic of PXE (left). Angioid streaks in the eye (right). Reproduced from Li et al., Mutation Analysis (ABCC6) in a Family with Pseudoxanthoma Elasticum: Presymptomatic Testing with Prognostic Implications, Oxford University Press, 2010, 163, 2, 641-3, by permission of Oxford University Press [16]

Supplemental Table 1 contains patient-level genotype and phenotype information, which demonstrates heterogeneity in the clinical presentation of pediatric ABCC6 deficiency at the individual level. Whereas some patients presented with a classic GACI2 phenotype, and others presented with characteristic features of PXE, there were many patients who exhibited a phenotype that did not fit squarely within these diagnostic categories, including those with severe neurologic problems or pediatric-onset cardiovascular disease, with or without skin or ocular findings. To illustrate this phenotypic spectrum, four patient cases (included in this cohort) have been highlighted in Fig. 3.

### Genotypes & genotype-phenotype correlations

#### *ABCC6* variants and pathogenicity

**Fig. 4:**
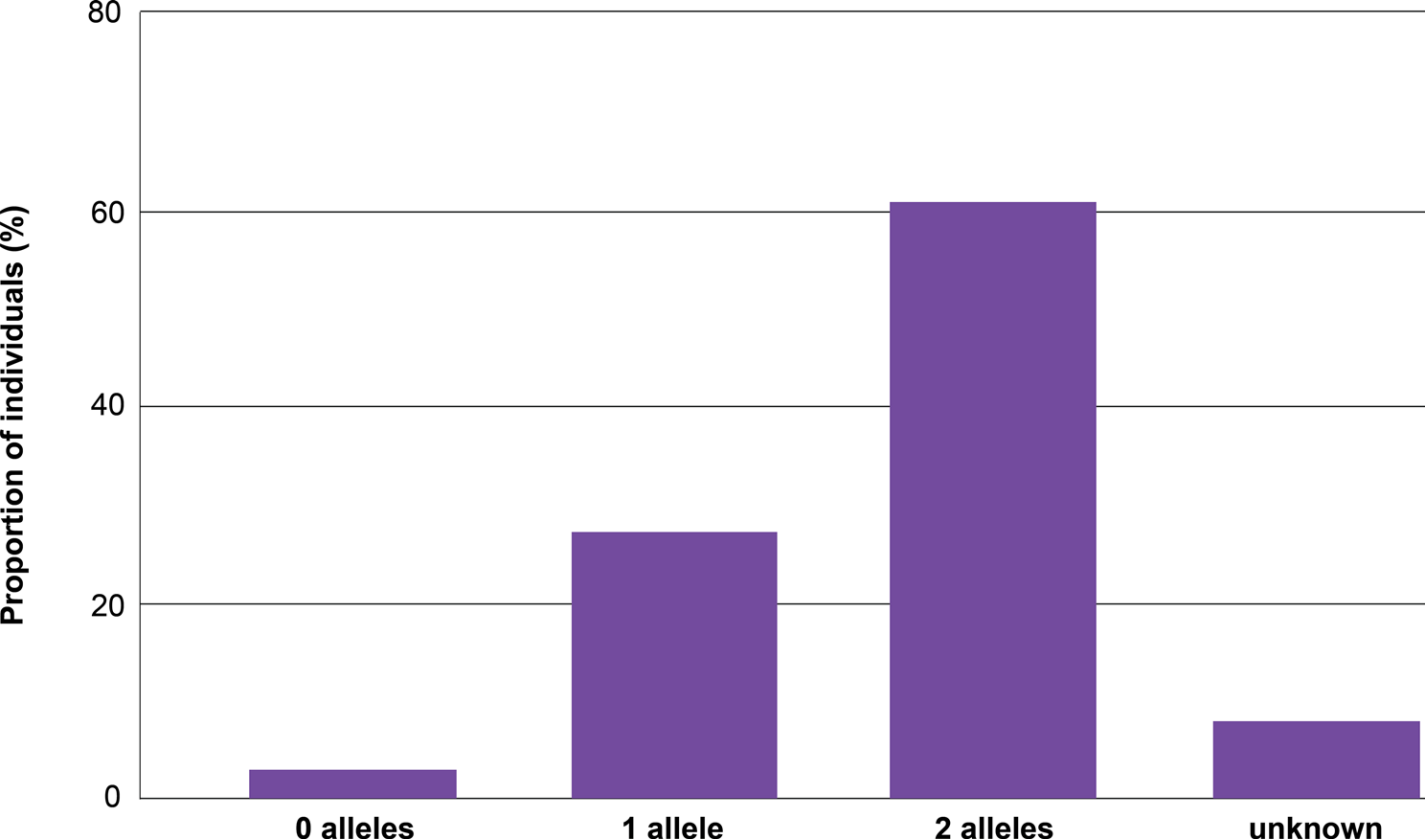
Individuals by number of reported P/LP *ABCC6* alleles

**Fig. 5:**
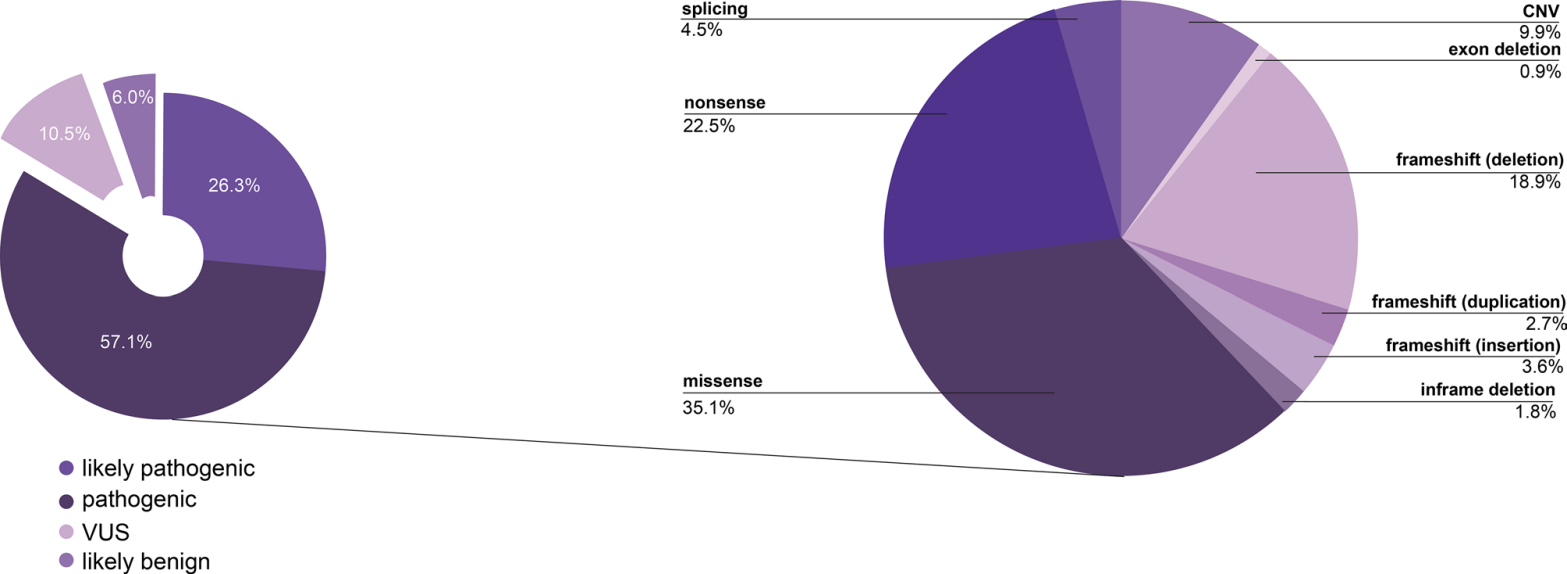
**A**) Pathogenicity call of all unique variants excluding unknown (*N* = 133). **B**) Distribution of P/LP variants (*n* = 111) by variant type

A total of 133 unique *ABCC6* variants were identified. The majority of individuals (61.0%) had two identified *ABCC6* variants, 27.4% had one variant, and 11.6% had no or unknown variants (i.e., a published diagnosis of ABCC6 deficiency without the variant specified; Fig. 4). Of these, 57.1% (76/133) of variants were pathogenic (P), 26.3% (35/133) were likely pathogenic (LP), 10.5% (14/133) were variants of uncertain significance, and 5.3% (7/133) were likely benign. ABCC6 variants were distributed across the protein (Fig. 7). Among the P/LP variants, the most common were missense (35.1%), nonsense (22.5%), and frameshift caused by a deletion (18.9%) (Fig. 5).

Among the 95 individuals included in this analysis, eight also had monoallelic variants in another gene. These included *ENPP1* (*n* = 4), *NOMO3* (*n* = 2), *GGCX* (*n* = 1), and *CBS* (*n* = 1).

#### Clinical phenotype by zygosity

**Fig. 6:**
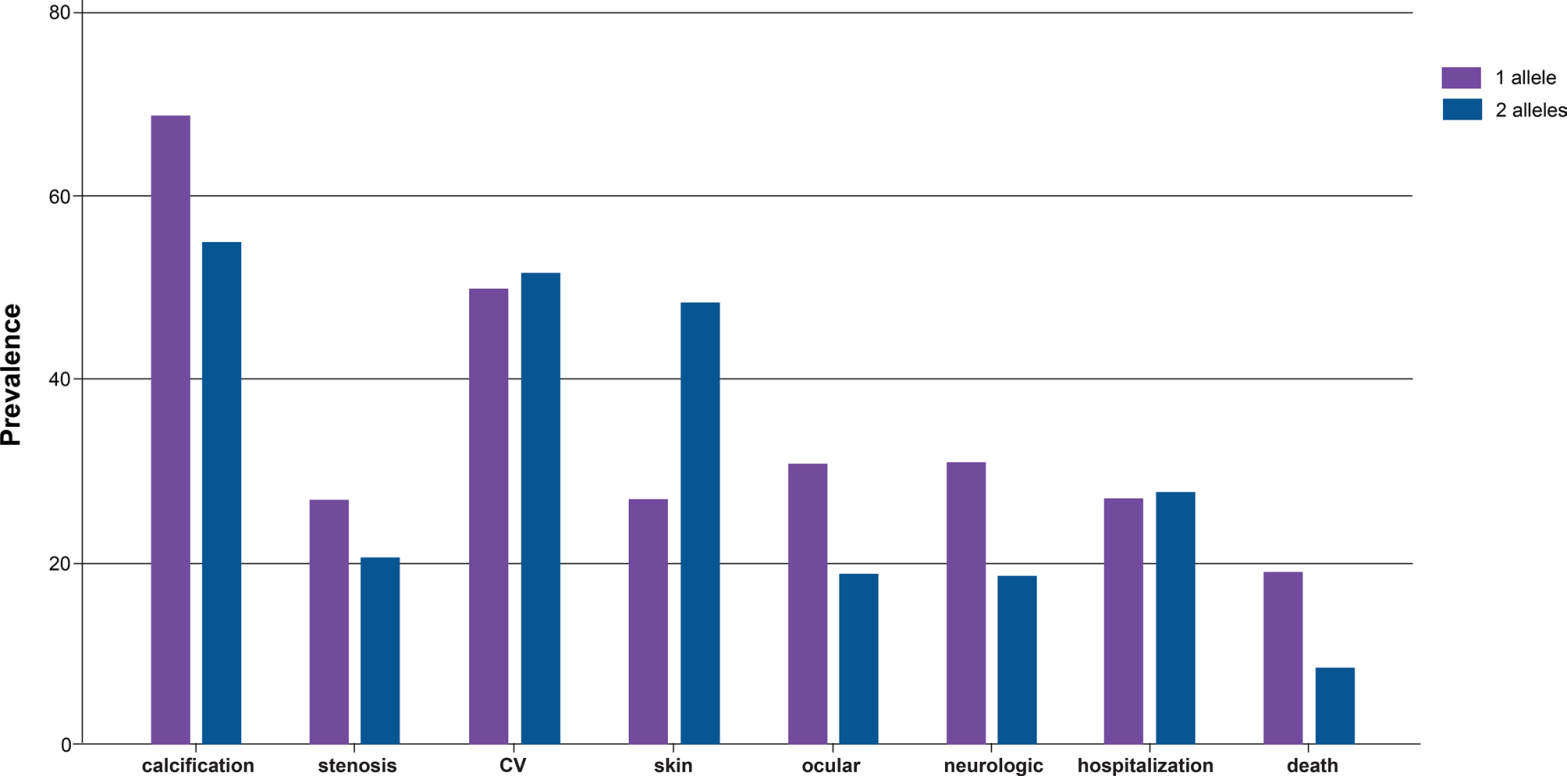
Phenotypes by number of reported *ABCC6* variants. Bar graph depicting prevalence of calcification, stenosis, cardiovascular (CV), skin, ocular, neurologic, hospitalization, and death in individuals with one versus two *ABCC6* variants. Presented as percentages of individuals with one (*n* = 26) and two variants (*n* = 58)

Biallelic *ABCC6* variants were observed in 71.4% of patients diagnosed with PXE, compared to 52.8% of patients diagnosed with GACI, while the prevalence of one identified *ABCC6* variant was more similar across diagnoses (21.4% vs. 32.1%) (Table 1). Clinical manifestations differed between individuals with one (*n* = 26) and two identified *ABCC6* variants (*n* = 58) (Fig. 6). A higher percentage of individuals with biallelic variants had skin manifestations, while calcification and neurologic and ocular manifestations were relatively more prevalent among individuals with one identified variant. Surprisingly, a higher prevalence of death was reported in individuals with a single identified *ABCC6* variant.

#### Phenotypes associated with most common *ABCC6* variants

**Fig. 7:**
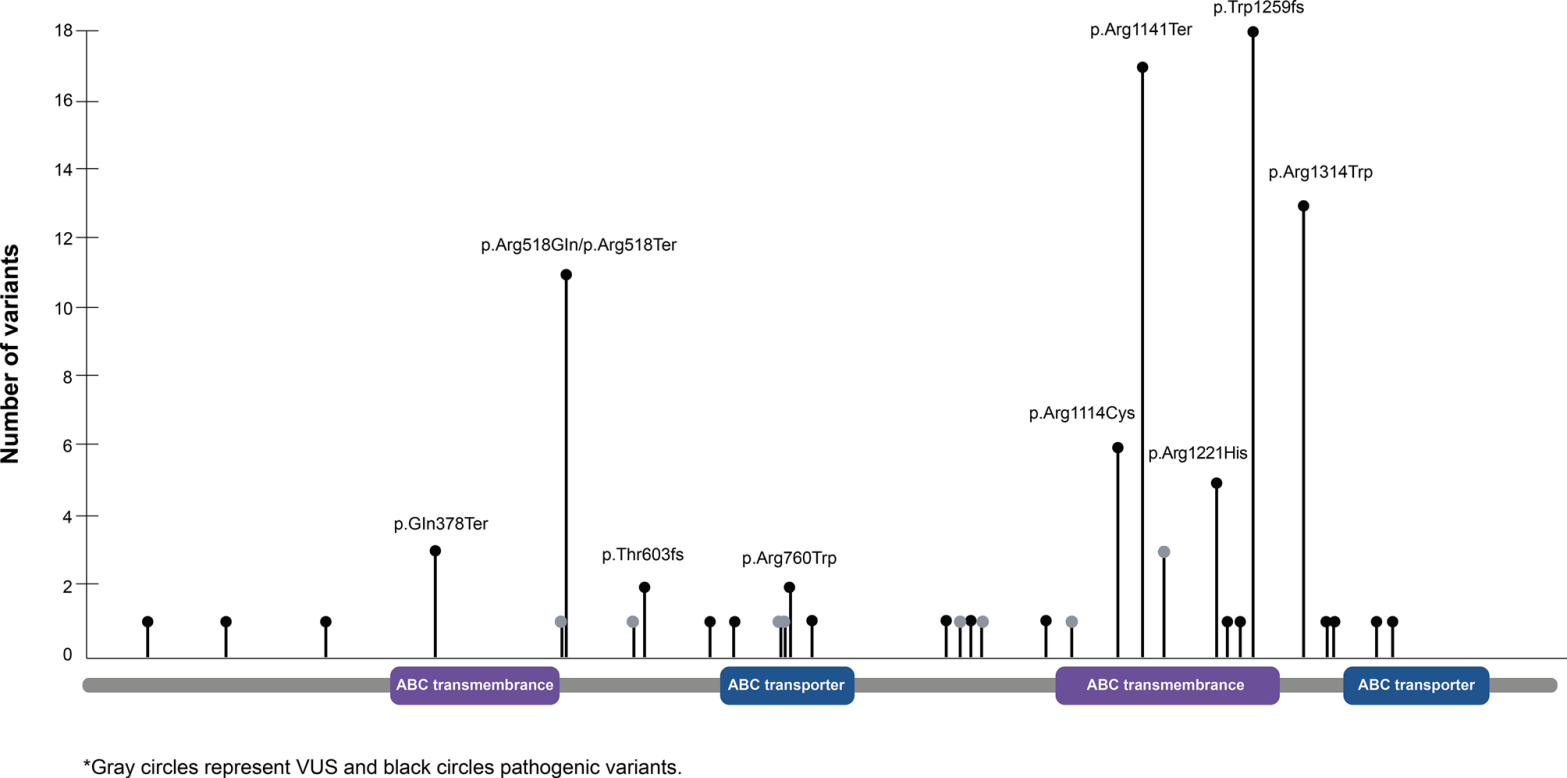
Number of ABCC6 protein variants by location in the protein sequence

Four *ABCC6* variants occurred most frequently in the analysis population (*n* > 10 variants), all of which were pathogenic (Fig. 7). Forty-three patients (45.3% of the population) carried one or more of these variants; their phenotypes are shown in Additional file 1, Table S1 and summarized in Additional file 2, Table S2. Ultimately, no genotype-phenotype correlation could be deduced. For example, the most common variant, p.Arg1141Ter, was identified in ten individuals diagnosed with GACI and six individuals diagnosed with PXE. The GACI cases presented in infancy with extensive cardiovascular calcification (*n* = 9), hypertension (*n* = 8), and heart failure (*n* = 6), while the PXE cases presented across a broad age range from infancy to age 16 years with heterogeneous neurologic, dermatologic, cardiovascular, and/or ocular manifestations of variable severity. Similar patterns were seen with the other most common variants (Additional files, Tables S1 and S2).

Of note, the second most common variant, p.Trp1259GlyfsTer14, was seen in ten patients. Nine of these patients were from a genetically isolated population in the Netherlands, and all had a homozygous genotype with PXE skin (8/9) and ocular (1/9) lesions. The tenth patient with a heterozygous p.Trp1259GlyfsTer14 variant was diagnosed with GACI and died from a myocardial infarction at age 22 days.

## Discussion

This study reports on a large cohort of pediatric patients with ABCC6 deficiency collected via literature search and two natural history studies. The primary aim was to elucidate the clinical and genetic features of this population and to evaluate potential genotype-phenotype correlations. Of the 95 individuals analyzed, 55.8% had a diagnosis of GACI2 and 44.2% had a diagnosis of PXE. Collectively, these children exhibited a high degree of soft tissue mineralization and vascular stenosis, with cardiovascular, dermatologic, ocular, and neurovascular impacts. Upon examining the ages at which clinical manifestations were recorded, we found that calcification and cardiovascular issues predominated in infancy, skin and ocular issues emerged after infancy, and neurologic problems were reported both in infants and young children. Skeletal anomalies observed in individuals with GACI2 were likely secondary to the use of bisphosphonates, which are known to be associated with skeletal toxicity.

**Fig. 8:**
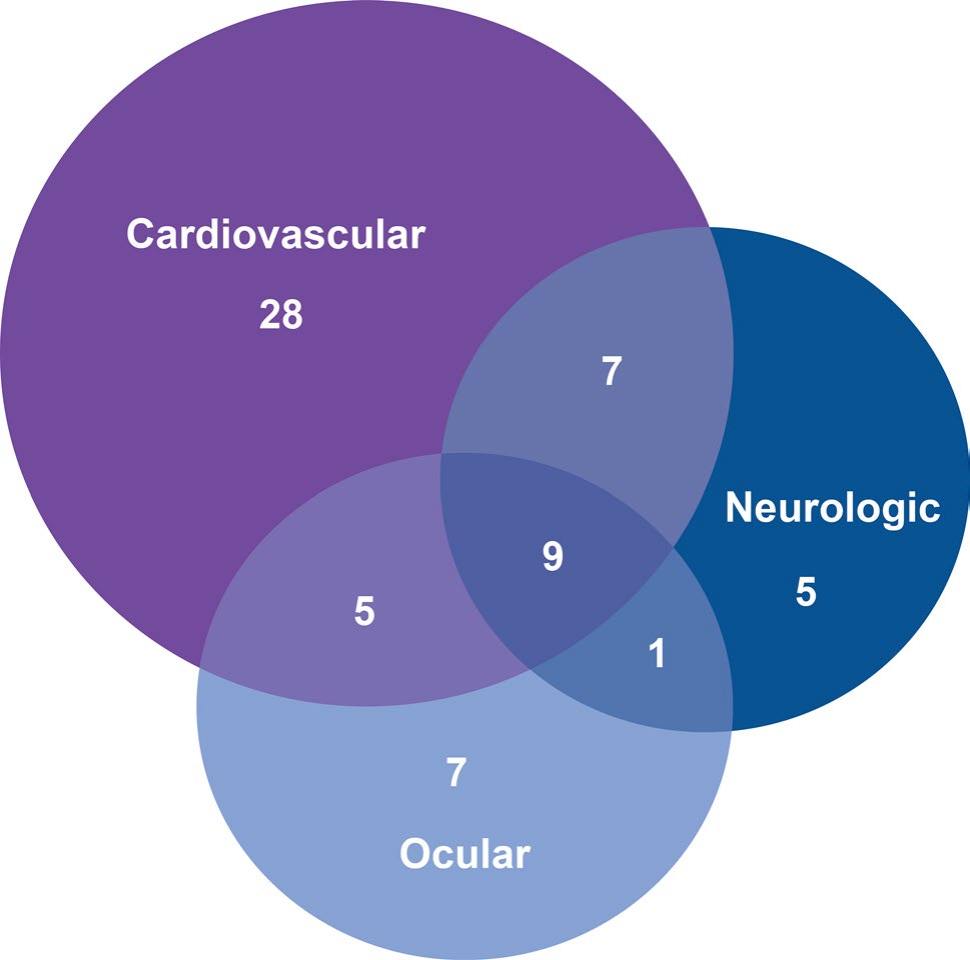
Number of patients with cardiovascular, neurologic, and/or ocular system involvement

At the individual level, these data show that ABCC6 deficiency can result in a spectrum of presentations across a range of ages, which may or may not fit the typical GACI2 or PXE phenotypes. Pediatric patients with ABCC6 deficiency had multiorgan involvement in various combinations (Fig. 8). Some individuals presented with a predominantly neurologic phenotype, which is not typically considered characteristic of GACI2 or PXE [4, 5, 10, 24, 36]. Our data included cases where individuals were not diagnosed until their skin manifestations became apparent, despite the existence of cardiovascular or neurologic complications at earlier ages (Fig. 2).

Within the cohort, there was a high degree of heterogeneity with regard to protein variant location and type. Patients with the four most common *ABCC6* variants (45.3% of the population) had significant variability in their clinical phenotypes, suggesting that an *ABCC6* genotype may not be able to predict the development of GACI vs. PXE. In this sample, 27.4% of individuals had a single identified *ABCC6* variant (21% deemed P/LP) and displayed a similar clinical phenotype to those with biallelic variants. The similarity of phenotypes points to the possibility that individuals with only one reported variant may instead have compound heterozygous *ABCC6* variants, with the missing variant on the second allele occurring in a deep intronic region. Digenic inheritance is also a possible explanation, though less likely. Eight individuals with other monoallelic gene variants in *ENPP1*,* NOMO3*,* GGCX*, and *CBS* genes were noted in this cohort, which may have had an impact on the phenotypes reported. This supports the theory that our understanding of ABCC6 expression remains incomplete, including the potential role of a yet-to-be-identified second *ABCC6* variant, and the interplay between other modifying variants.

Our study reflects the challenge of diagnosing ABCC6 deficiency in the pediatric population. Individuals collectively exhibited a spectrum of clinical phenotypes that overlap GACI2 and PXE, with no clear genotype-phenotype correlation. In addition, the median age of presentation of symptoms of PXE was earlier than generally expected [37]. This may result in diagnostic uncertainty, lack of a performance of genetic testing, and underdiagnosis of ABCC6 deficiency in the pediatric population. In most cases, a diagnosis was likely related to practitioners’ knowledge and awareness of the primary features of GACI2 or PXE, where lesser-appreciated characteristics, such as neurologic problems, may not prompt clinical suspicion.

A more precise definition of the features that may elevate the suspicion of ABCC6 deficiency can help establish clearer care pathways for patients. We confirm that a high index of suspicion should be given to the presentation of concurrent calcification with cardiovascular complications within the first year of life (Fig. 2). Beyond infancy, *ABCC6* genetic testing should be considered not only in those with characteristic cutaneous or ocular PXE lesions, but also in children presenting with stroke, seizure, or hypertension of undetermined etiology. Across the age spectrum, the identification of ectopic calcification or arterial stenosis on imaging should increase diagnostic suspicion for ABCC6 deficiency.

In addition to diagnostic education, there is a need for guidance on the initial evaluation and lifelong management of children newly diagnosed with ABCC6 deficiency. Although specific *ABCC6* variants may not allow for detailed prognoses, proactive monitoring can enable timely intervention for emerging clinical manifestations. A multidisciplinary care team including cardiology, neurology, ophthalmology, and genetics providers may be needed to screen for and manage relevant organ-system complications. At a minimum, we suggest that hypertension management, screening for cerebral vasculopathy, and secondary stroke prevention are appropriate considerations for this population.

This study provides an important view of the clinical presentation of ABCC6 deficiency in the pediatric population; however, ascertainment bias introduced through retrospective evaluation of patient cases should be taken into account. On one hand, children with a more severe phenotype may be over-represented in the published literature. On the other hand, as we included all children with a reported *ABCC6* genotype and any reported phenotypic information, many of the included cases (i.e. population genotype studies) were limited in the amount of clinical data that they reported. While some patients (i.e., those from the Natural History studies) were followed longitudinally, most cases included patient data from a single time point [12]. Likewise, as these patients were evaluated across many different international institutions, there was considerable variability across assessments conducted. For example, it is not clear how many patients underwent angiography for assessment of arterial stenosis, or neuropsychological testing for the presence of developmental delay. Limited duration of follow-up and lack of standardized assessments may have accounted for the relatively low prevalence of stroke observed in this study (9.4%) compared with a prior natural history study (21.0%) [12]. Together, these limitations suggest that the true burden of ABCC6 deficiency in the pediatric period is likely underestimated by this cohort.

## Conclusions

Infants and children with ABCC6 deficiency may present with a wide range of multiorgan complications, likely contributing to under recognition of this rare and often severe mineralization disorder. In this analysis, we characterized the genotypic and phenotypic presentation of ABCC6 deficiency in patients aged 0 to < 18 years, revealing a broad spectrum of cardiovascular, dermatologic, ocular, and neurologic signs and symptoms, with individuals exhibiting phenotypes along the spectrum of GACI2 and PXE. These findings underscore the urgent need for improved education and awareness of pediatric ABCC6 deficiency among the medical community to support timely diagnosis and refine screening and management recommendations.

## Supplementary Information

Below is the link to the electronic supplementary material.

Supplementary Material 1: **Table S1** ABCC6 Patient Database (Additional file 1 table S1.xlsx). All patients (N=95) and their ABCC6 genotype, demographics, diagnosis, survival outcome, and published phenotypes. ACA=anterior cerebral artery; AIS=arterial ischemic stroke; CNS=central nervous system; F=female; GACI= Generalized arterial calcification of infancy; HIE=Hypoxic ischemic encephalopathy; ICA=internal carotid artery; IVC=inferior vena cava; LCA=left main coronary artery; LA=left atrium; LV=left ventricle; M=male; MCA=middle cerebral artery; PCA=posterior cerebral artery; PDA=patent ductus arteriosus; PFO=patent foramen ovale; PICA=posterior inferior cerebellar artery; PPHN=persistent pulmonary hypertension of the newborn; PXE=pseudoxanthoma elasticum; RCA=right coronary artery; RV=right ventricle; VUS=variant of uncertain significance

Supplementary Material 2: **Table S2** Phenotypes associated with the four most frequently recorded variants

## Data Availability

The data that support the findings of this analysis are not openly available due to reasons of sensitivity and are available from the corresponding author upon reasonable request.

## Abbreviations

ABCC6: ATP binding cassette subfamily C member 6
ABI: Ankle Brachial Index
ACMG: American College of Medical Genetics and Genomics
ACMG/AMP: American College of Medical Genetics and Genomics and Association for Molecular Pathology
AMP: Adenosine monophosphate
ARHR2: Autosomal recessive hypophosphatemic rickets type 2
ATP: Adenosine triphosphate
CNV: Copy number variation
CV: Cardiovascular
DSA: Digital subtraction angiography
ENPP1: Ectonucleotide pyrophosphatase-phosphodiesterase family member 1
GACI: Generalized arterial calcification of infancy
GACI1: Generalized arterial calcification of infancy Type 1
GACI2: Generalized arterial calcification of infancy Type 2
ICA: Internal carotid artery
LP: Likely pathogenic
NICU: Neonatal Intensive Care Unit
NIH: National Institute of Health
P: Pathogenic
PAD: Peripheral artery disease
P/LP: Pathogenic/Likely pathogenic
P_i_: Inorganic phosphate
PP_i_: Inorganic pyrophosphate
PXE: Pseudoxanthoma elasticum
TIA: Transient ischemic attack
VUS: Variant of uncertain significance
