# Supplementary material for: Pediatric ABCC6 deficiency: a genotypic and phenotypic analysis
Source: Orphanet J Rare Dis. 2025 Nov 19;20:593. doi: 10.1186/s13023-025-04102-7 (PMC12628958; doi:10.1186/s13023-025-04102-7)
Supplement: Supplementary file 2 — Supplementary Material 2: Table S2 Phenotypes associated with the four most frequently recorded variants [file 13023_2025_4102_MOESM2_ESM.docx]

**Additional file 1, Table S1.** ABCC6 Patient Database (Additional file 1 table S1.xlsx). All patients (N = 95) and their *ABCC6* genotype, demographics, diagnosis, survival outcome, and published phenotypes.

ACA = anterior cerebral artery; AIS = arterial ischemic stroke; CNS = central nervous system; F = female; GACI = Generalized arterial calcification of infancy; HIE = Hypoxic ischemic encephalopathy; ICA = internal carotid artery; IVC = inferior vena cava; LCA = left main coronary artery; LA = left atrium; LV = left ventricle; M = male; MCA = middle cerebral artery; PCA = posterior cerebral artery; PDA = patent ductus arteriosus; PFO = patent foramen ovale; PICA = posterior inferior cerebellar artery; PPHN = persistent pulmonary hypertension of the newborn; PXE = pseudoxanthoma elasticum; RCA = right coronary artery; RV = right ventricle; VUS = variant of uncertain significance.

**Additional file 2, Table S2.** Phenotypes associated with the four most frequently recorded variants.

|  | **Zygosity** | | | **Survival Outcome** | | | **Diagnosis** | | **Clinical phenotypes** | | | | |
| --- | --- | --- | --- | --- | --- | --- | --- | --- | --- | --- | --- | --- | --- |
|  | **Homozygous** | **Compound heterozygous** | **Single allele** | **Alive** | **Dead** | **Unknown** | **PXE** | **GACI2** | **Ocular** | **Cardiovascular** | **Neurologic** | **Calcification** | **Hospitalizations** |
| p.Trp1259GlyfsTer14 | 9 | 0 | 1 | 9 | 1 | 0 | 9 | 1 | 1 | 1 | 0 | 1 | 1 |
| p.Arg1141Ter | 1 | 12 | 3 | 12 | 2 | 2 | 6 | 10 | 1 | 3 | 11 | 12 | 8 |
| p.Arg1314Trp | 4 | 3 | 2 | 6 | 2 | 1 | 1 | 8 | 3 | 8 | 3 | 8 | 3 |
| p.Arg518Gln or p.Arg518Ter | 3 | 5 | 0 | 4 | 0 | 1 | 3 | 5 | 1 | 3 | 0 | 4 | 2 |
